# Collectanea Medica: Consisting of Anecdotes, Facts, Extracts, Illustrations, &c.

**Published:** 1820-06

**Authors:** 


### COLLECTANEA MEDICA:

CONSISTING OF

### ANECDOTES, FACTS, EXTRACTS, ILLUSTRATIONS, &c.

*Relating to the History or the Art of Medicine, and the Auxiliary Sciences.*

Floriferis ut apes in saltibus omnia libant,
Omnia nos itidem depascimur aurea dicta.

### *Observations on the Qualities of Wines.*

[From Dr. PARIS's *Pharmacologia.*]

THE term wine is more strictly, and especially, applied to express the fermented juice of the grape, although it is generally used to denote that of any sub-acid fruit. The presence of tartar is, perhaps, the circumstance by which the grape is most strongly distinguished from all the other sub-acid fruits that have been applied to the purpose of wine-making: the juice of the grape, moreover, contains within itself all the principles essential to vinification, in such a proportion and state of balance, as to enable it at once to undergo a regular and complete fermentation; whereas, the juices of other fruits require artificial additions for this purpose; and the scientific application and due adjustment of these means, constitute the art of making wines.* It has been remarked, that all those wines that contain an excess of malic acid, are of a bad quality: hence the grand defect that is necessarily inherent in the wines of this country, and which leads them to partake of the properties of cider; for, in the place of the tartaric, the malic acid always predominates in our native fruits.

The characteristic ingredient of all wines is alcohol; and the quantity of this, and the condition or state of combination in which it exists, are the circumstances that include all the interesting and disputed points of medical enquiry. Daily experience convinces us that the same quantity of alcohol, applied to the stomach under the form of natural wine, and in a state of mixture with water, will produce very different effects upon the body, and to an extent which it is difficult to comprehend: it has, for instance, been demonstrated, that port, madeira, and sherry, contain from one-fourth to one-fifth their bulk of alcohol, so that a person who takes a bottle of either of them, will thus take nearly half-a-pint of alcohol, or almost a pint of pure brandy! and, moreover, that different wines, although of the same specific gravity, and consequently containing the same absolute proportion of spirit, will be found to vary very considerably in their intoxicating powers. No wonder, then, that such results should stagger the philosopher, who is naturally unwilling to accept any tests of difference from the nervous system which elude the ordinary resources of analytical che-

* For an account of which, the reader is referred to a most ingenious and interesting essay, by Dr. MACCULLOCH, entitled *Remarks on the Art of making Wine, with Suggestions for the Application of its Principles to the Improvement of Domestic Wines.*

mistry. The conclusion was therefore drawn, that alcohol must necessarily exist in wine in a far different condition from that in which we know it in a separate state; or, in other words, that its elements only could exist in the vinous liquor, and that their union was determined, and consequently alcohol produced, by the action of distillation. That it was the product, and not the educt, of distillation, was an opinion which originated with ROUELLE, who asserted that alcohol was not completely formed until the temperature was raised to the point of distillation. More lately, the same doctrine was revived and promulgated by FABBRONI, in the memoirs of the Florentine Academy. GAY-LUSSAC has however silenced the clamorous partisans of this theory, by separating the alcohol by distillation at the temperature of 66° Fah.; and, by the aid of a vacuum, it has since been effected at 56°. Besides, it has been shown that, by precipitating the colouring matter, and some of the other elements of the wine, by sub-acetate of lead, and then saturating the clear liquor with sub-carbonate of potass, the alcohol may be completely separated without any elevation of temperature; and, by this ingenious expedient, Mr. BRANDE has been enabled to construct a table, exhibiting the proportions of combined alcohol which exist in the several kinds of wine. No doubt, therefore, can remain upon this subject; and the fact of the difference of effect produced by the same bulk of alcohol, when presented to the stomach in different states of combination, adds another striking and instructive illustration to those already enumerated in the course of this work, of the extraordinary powers of chemical combination in modifying the activity of substances upon the living system. In the present instance the alcohol is so combined with the extractive matter of the wine, that it is probably incapable of exerting its full specific effects upon the stomach before it becomes altered in its properties, or, in other words, digested: and this view of the subject may be fairly urged in explanation of the reason why the intoxicating effects of the same wine are so liable to vary in degree in the same individual, from the peculiar state of his digestive organs at the time of its potation. Hitherto we have only spoken of pure wine; but it is essential to state, that the stronger wines of Spain, Portugal, and Sicily, are rendered marketable in this country by the addition of brandy, and must consequently contain uncombined alcohol, the proportion of which, however, will not necessarily bear a ratio to the quantity added, because, at the period of its admixture, a renewed fermentation is produced by the scientific vintner, which will assimilate and combine a certain portion of the foreign spirit with the wine: this manipulation, in technical language, is called *fretting-in*. The free alcohol may, according to the experiments of Fabbroni, be immediately separated, by saturating the vinous fluid with sub-carbonate of potass, while the combined portion will remain undisturbed. In ascertaining the fabrication and salubrity of a wine, this circumstance ought always to constitute a leading feature in the enquiry; and the tables of Mr. Brande would have been greatly enhanced in practical value, had the relative proportions of uncombined spirit been appreciated in his experiments; since it is to this, and not to the combined alcohol, that the injurious effects of wine are to be attributed. "It is well known," observes Dr. MACCULLOCH, "that diseases of the liver are the most common, and the most formidable, of those produced by the use of ardent spirits: it is equally certain, that no such disorders follow the intemperate use of pure wine, however long indulged in. To the concealed and unwitting consumption of spirit, therefore, as contained in the wines commonly drank in this country, are to be attributed the excessive prevalence of those hepatic affections which are, comparatively, little known to our continental neighbours." Thus much is certain, that their ordinary wines contain no alcohol, but that which is disarmed of its virulence by the prophylactic energies of combination.

The odour or *bouquet*, and flavour, which distinguish one wine from another, evidently depend upon some volatile and fugacious principle, soluble in alcohol. This, in sweet and half-fermented wines, is immediately derived from the fruit, as in those from the Frontignac and Muscat grapes; but, in the more perfect wines, as in claret, hermitage, rivesaltes, and burgundy, it bears no resemblance to the natural flavour of the fruit, but is altogether the product of the vinous process; and, in some wines, it arises from the introduction of flavouring ingredients, as from almonds in Madeira wines, as well as in those of Xeres and Saint Lucar, and hence their well-known nutty flavour. Among the ancients it was formerly, and in modern Greece it is to this day, the fashion to give a resinous flavour by the introduction of turpentine into the casks.

Wines admit of being arranged into four classes:

1. *Sweet wines;* which contain the greatest proportion of extractive and saccharine matter, and generally the least ardent spirit, though this is often rather disguised than absent. As in these wines a proportion of sugar has remained unchanged during the process of vinification, they must be considered as the results of an imperfect fermentation, and are, in fact, mixtures of wine and sugar: accordingly, whatever arrests the progress of fermentation must have a tendency to produce a sweet wine. Thus, boiling the *must*, or drying the fruit, will, by partially separating the natural leaven and dissipating the water, occasion such a result as is exemplified by the manufacture of the wines of Cyprus, the *vino cotto* of the Italians, and the *vinum coctum* of the ancients, by that of Frontignac, the rich and luscious wines of Canary, the celebrated *tokay*, *vino tinto* (tent of Hungary), the Italian *montefiascone*, the Persian *schiras*, the *malmsey* wines of Candia, Chio, Lesbos, and Tenedos, and those of the other islands of the Archipelago. The wines of the ancients, as CHAPTAL observes, were so concentrated by boiling, that they rather deserve the name of extracts or syrups than that of wines; they must have been very sweet, and but little fermented. Apparently to remedy this, they were kept for a great length of time: according to ARISTOTLE and GALEN, seven years was the shortest period necessary for keeping wine before it was fit to drink; but wines of a century old were not uncommon at the tables of the luxurious citizens of ancient Rome: and HORACE boasts of his drinking *falernian*, born as it were with him, or which reckoned its age from the same consuls.

2. ***Sparkling or effervescing wines***, as champagne, are indebted for their characteristic properties to the presence of carbonic acid. They rapidly intoxicate, in consequence of the alcohol, which is suspended in, or combined with, this gas, being thus applied in a sudden and very divided state to a large extent of nervous surface: for the same reason, their effects are as transitory as they are sudden.

3. ***Dry and light***. These are exemplified by the more esteemed German wines, as *hock*, *Rhenish*, *Mayne*, *Moselle*, *Necker*, and *Elsass*, and those highly-flavoured wines, *Burgundy*, *Claret*, *Hermitage*, &c. They contain a very inconsiderable degree of ardent spirit, and combine with it the effect of an acid.

4. ***Dry and strong***, as *madeira, port, sherry*, &c. The name *sec*, corruptly written sack, signifies dry; the sec wine prepared at Xeres* in Spain, is called, according to our orthography, *sherris*, or sherry. In the manufacture of sherry, lime† is added to the grapes; a circumstance, observes Dr. Macculloch, apparently conducive to its well-known dry quality, and which probably acts by neutralizing a portion of malic or tartaric acid.

By the adulteration or medication of wines, three principal objects are attempted, viz. 1. To give them strength, which is effected by adding any ardent spirit; but the wine is slowly decomposed by it. 2. To perfect or change their colour. It is very usual to change white wines, when they have grown brown or rough, into red wines, by means of sloes or other colouring matter. 3. To lessen or remove their acidity. It is well known that lead in different forms has frequently been employed for this purpose: the practice, however, is attended with the most dangerous consequences; but which Dr. Macculloch is inclined to believe has been over-rated, since the compounds which this metal forms with the tartaric and malic acids are insoluble: but against this argument the decisive results of experience may be opposed; and Fourcroy conceived that, by the addition of lead, a soluble triple salt, an aceto-tartrate of lead, was produced. The fraud may be easily detected by the test‡ invented by Dr. Hahnemann. The ancients, it appears, were acquainted with this property in lead; for, according to Pliny, the Greeks and Romans improved the quality of their wines by immersing a plate of lead in them.§ Wine, as a pharmaceutical agent, is employed to extract several of the principles

---

* ξηρος signifies *dry*. This is a curious coincidence.

† The *sack* of Shakspeare was probably *sherry*; a conjecture which receives additional strength from the following passage:

*Falstaff*.—"You rogue, here's *lime* in this sack too: there is nothing but roguery to be found in villainous man: yet a coward is worse than a cup of sack with *lime* in it; a villainous coward."

‡ Expose equal parts of sulphur and powdered oyster-shells to a white heat for fifteen minutes, and, when cold, add an equal quantity of cream of tartar: these are to be put into a strong bottle with common water, to boil for an hour; and the solution is afterwards to be decanted into ounce phials, adding twenty drops of muriatic acid to each. This liquor will precipitate the least quantity of lead from wines in a very sensible black precipitate. As iron might be accidentally contained in the wine, the muriatic acid is added to prevent its precipitation.

§ Lead will not only correct the acidity of wines, but remove the rancidity of oils; a property which is well known to painters, and which affords an expedient for making an inferior olive-oil pass for good.

of vegetables, and to dissolve certain mineral bodies. As a solvent, however, it is liable to many serious objections, as inequality of strength and uncertainty of composition: thus, sound and perfectly-fermented dry wine, as sherry, is frequently unable to dissolve iron, while tartarized antimony is instantly decomposed by every other. As a menstruum to obtain an extract, it is quite inadmissible, on account of the residuum which it leaves by evaporation.

---

### *Observations on the Properties and Medicinal Use of Elaterium.*

[From the same.]

This substance spontaneously subsides from the juice of the wild cucumber, in consequence, I presume, of one of those series of changes which vegetable matter is perpetually undergoing, although we are hitherto unable to express them by any known chemical law. It is therefore not an extract, either in the chemical or pharmaceutical acceptation of the term, nor an inspissated juice, nor is it a *fecula,** as it has been termed: the Dublin College has perhaps been most correct in simply calling it Elaterium, the name given to it by Dioscorides.

It occurs, in commerce, in little thin cakes or broken pieces, bearing the impression of the muslin upon which it was dried. Its colour is greenish, its taste bitter and somewhat acrid; and, when tolerably pure, it is light, pulverulent, and inflammable.

The early history of this medicinal substance is involved in great perplexity, each author speaking of a different preparation by the same name: for instance, the elaterium of Dioscorides must have been a very different substance from that of Theophrastus; and, wherever Hippocrates mentions the term, he evidently alludes to *any* violent purgative. "Hippocrati elaterium medicamentum est quod per alvum expurgat." (*Bod. in Theophrast.*) This will in some degree reconcile the discordant testimonies of different authors with regard to the powers of elaterium: for example, Dioscorides states its dose to be from grs. ii to ℈j; in Ætius, Paulus, and Actuarius, it is recommended to the extent of ʒss; in Mesue, from ℈ss to ℈j; in Bontius (*Med. Ind.*) from ℈j to ʒss; Massarias exhibits it in doses of grs. vj; Fernelius and Senneretus, to ℈j; Herman, from grs. v to vij; Quincy, to grs. v; and Boerhaave does not venture to give more than grs. iv; and the practitioners of the present day limit their dose from gr. ½ to grs. ij. Dr. Clutterbuck, with a laudable intention to discover some method of procuring this article at a cheaper rate, and at the same time of discovering some process which might ensure a preparation of more uniform strength, has lately performed a series of interesting and instructive experiments;† the results of which prove, in a satisfactory

---

* The juices of iris-root, arum-root, and bryony-root, and those of many other plants, allow their medicinal elements to separate and subside in a similar manner, leaving the super-natant liquid perfectly inert: if we must have a generic name to express such substances, it should be termed a *feculence*, rather than a *fecula*.

† *Observations on the Nature and Preparation of the Elaterium*, read at the Medical Society of London, April 24, 1819; and which were published in the *Medical Repository*, vol. xiii. No. 67.

manner, "that the active principle of this plant is neither lodged in the roots, leaves, flowers, or stalks, in any considerable quantity; nor is it to be found in the body of the fruit itself, or in the seeds, but in the juice around the seeds:" the substance which spontaneously subsides from this liquor, obtained without pressure, is genuine elaterium, the quantity of which contained in the fruit is extremely small; for Dr. Clutterbuck obtained only six grains from forty cucumbers. This gentleman communicated the detail of these experiments to the president of the College of Physicians, who requested me, as Professor of materia medica, to report upon them. I accordingly deemed it to be my duty to enter upon a series of new experiments, which I have lately completed, with the able assistance of Mr. Farraday, in the laboratory of the Royal Institution; the results of which will show, that, although Dr. Clutterbuck found that an eighth part of a grain of elaterium seldom failed to purge violently, yet, strange as it may appear, that not more than one grain in ten of elaterium, as it occurs in commerce, possesses any active properties; and that this decimal part is a vegetable proximate principle, not hitherto noticed, to which I shall give the name of *Elatin.* I shall subjoin the detail of my experiments in a note;* and I think it will appear, that their results will

---

* PROXIMATE ANALYSIS OF ELATERIUM.

*Experiments. Series 1st.*

A. Ten grains of elaterium, obtained from a respectable chemist, and having all the sensible properties which indicated it to be genuine, were digested, for twenty-four hours, with distilled water, at a temperature far below that of boiling: *four grains* only were dissolved.

B. The solution was intensely bitter; of a brownish-yellow colour; and was not in the least disturbed by alcohol, although a solution of iodine produced a blue colour: the solution therefore contained no gum, and only slight traces of starch.

C. The solution, after standing twenty-four hours, yielded a pellicle of insoluble matter, which, when burnt, appeared to resemble gluten.

D. The six grains which were insoluble in water were treated, for forty-eight hours, with alcohol of the specific gravity ·817, at 66° of Fahrenheit: a green solution was obtained; but, by slow evaporation, only half a grain of solid green matter was procured. The insoluble residue obstinately adhered to, and coated, the filtre like a varnish, and completely defended the mass from the action of the alcohol: it is probable that it consisted principally of fecula.

*Experiments. Series 2d.*

E. Ten grains of elaterium, from the same sample, were treated with alcohol of the specific gravity ·817, at 66° Fah. for twenty-four hours: upon being filtered, and the residuum washed with successive portions of alcohol, the elaterium was found to have lost only 1·6 of a grain. The high specific gravity of the alcohol in this experiment was important: had it been lower, different results would have been produced.

F. The alcoholic solution, obtained in the last experiment, was of a most brilliant and beautiful green colour, resembling that of the oil of cajeput, but brighter; upon slowly evaporating it, 1·2 grains of solid green matter was obtained.

G. The solid green matter of the last experiment was treated with boiling distilled water; when a minute portion was thus dissolved, and a solution of a most intensely bitter taste, and of a brownish-yellow colour, resulted.

H. The residue, insoluble in water, was inflammable, burning with smoke and an aromatic odour, not in the least bitter: it was soluble in alkalies, and was again precipitated from them unchanged in colour. It formed, with pure alcohol, a beautiful tincture, which yielded an odour of a very nauseous kind, but of very authorize me to express the chemical composition of elaterium in the following manner:

| | | | |
|---|---|---|---|
| | F. | Water | ·4 |
| I. | B. | Extractive | 2·6 |
| I. | B.D.J. | Fecula | 2·8 |
| I. | C. | Gluten | ·5 |
| | K. | Woody matter | 2·5 |
| | H. | *Elatin* | 1·2 (H and G together) |
| | G. | Bitter principle | |
| | | | 10 grains. |

It appears that the whole of the elatin does not separate itself from its native juice by spontaneous subsidence, and that, on this account, the supernatant liquor possesses some powers as a cathartic. We cannot be surprised, therefore, that the elaterium of commerce should be a very variable and uncertain medicine; for, independent of the great temptation which its high price holds out for adulterating it, which is frequently done with starch, it necessarily follows, that, where the active principle of a compound bears so small a proportion to its bulk, it is liable to be affected by the slightest variation in the process for its preparation, and even by the temperature of the season. Where pressure is used for obtaining the juices, a greater or less quantity of the inactive parts of the cucumber will be mixed with the elatin, in proportion to the extent of such pressure; and the elaterium will, of course, be proportionally weak.* There is one curious result obtained in my experiments, which deserves notice, viz. that there is a bitter principle in the elaterium, very distinct from its extractive matter, and totally unconnected with its activity; for I diluted the solution obtained in experiment G, and swallowed it, but it produced upon me no effect, except that which I generally experience upon taking a powerful bitter,—an increased appetite. The solution B was given to a person, but no effect whatever ensued.

Dose of good elaterium, as it occurs in commerce, is about two grains; or it is better to give it only to the extent of half a grain at a time, and to repeat that dose every hour until it begins to operate. It is probably, when thus managed, the best hydragogue cathartic which we possess.

---

little flavour, and which gave a precipitate with water: it was soft, and of considerable specific gravity, sinking rapidly in water; circumstances which distinguish it from common resin. In very minute quantities, it purges. It appears to be the element in which all the powers of the elaterium are concentrated, and which have been denominated *Elatin.*

I. The residuum, insoluble in alcohol, weighing 8·4 grs. (*Expt.* E.) was boiled in double-distilled water, when 5·9 grs. were dissolved.

J. The above solution was copiously precipitated blue by a solution of iodine, and was scarcely disturbed by the per-sulphate of iron.

K. The part insoluble, both in alcohol and water, which was left after Experiment I. amounted to 2·5 grains: it burnt like wood, and was insoluble in alkalies.

* When it has a dark-green colour, approaching to black, is compact, and very heavy, and breaks with a shining resinous fracture, we may reject it as an inferior article.

